# To the Patrons of the Medical Examiner

**Published:** 1848-12

**Authors:** 


### THE MEDICAL EXAMINER.

PHILADELPHIA, DECEMBER, 1848.

### TO THE PATRONS OF THE MEDICAL EXAMINER.

The present number of the Medical Examiner completes the volume for the current year. Eleven years have now elapsed since the journal was commenced, during the last five of which it has been conducted by the present editor. On entering upon the task, he declared it to be his determination to conduct the journal on broad and liberal principles, and on no account to admit anything to its columns which could be regarded as at all personal or unprofessional, and to this resolution he has constantly adhered, under all circumstances of provocation ; and such will ever be his conduct as a journalist. That his course in this respect is approved by the profession, he has the strongest evidence in the constantly increasing number of subscribers to the publication. This gratifying fact encourages the belief, too, that his efforts to render its pages interesting and acceptable have been successful, notwithstanding the small share of his time and attention which it has been possible to spare to it from other pressing engagements. Hereafter, he has reason to hope for amendment in this respect, and that the pages of the Examiner will be found still more worthy of the liberal support accorded to it by the profession.

